# Pyruvate Kinase M2 Mediates Glycolysis Contributes to Psoriasis by Promoting Keratinocyte Proliferation

**DOI:** 10.3389/fphar.2021.765790

**Published:** 2021-10-18

**Authors:** Yun-zi Liu, Ming-yuan Xu, Xiao-yu Dai, Lang Yan, Lei Li, Rui-zhen Zhu, Li-jun Ren, Ji-qian-zhu Zhang, Xiao-fang Zhang, Jin-feng Li, Yi-jun Tian, Wen-jing Shi, Ye-qiang Liu, Chun-lei Jiang, Jiang-bo Zhu, Ji-kuai Chen

**Affiliations:** ^1^ Department of Health Toxicology, Faculty of Naval Medicine, Second Military Medical University, Shanghai, China; ^2^ Laboratory of Stress Medicine, Faculty of Psychology and Mental Health, Second Military Medical University, Shanghai, China; ^3^ Department of Dermatopathology, Shanghai Skin Disease Hospital Affiliated to Tongji University, Shanghai, China; ^4^ The Second Naval Hospital of the Southern Theater of the Chinese People’s Liberation Army, Hainan, China

**Keywords:** pyruvate kinase M2, psoriasis, glycolysis, keratinocyte, shikonin

## Abstract

Psoriasis is characterized by keratinocyte proliferation and immune cell infiltration. M2 isoform of pyruvate kinase (PKM2) was reported to have an important role in cell proliferation, which is a rate-limiting enzyme that regulates the final step of glycolysis. However, how PKM2 regulates cell metabolism and proliferation in psoriatic keratinocytes is still poorly understood. Interestingly, we found that PKM2 was highly expressed in psoriatic epidermis from patients and mouse models. PKM2 overexpression promoted keratinocyte glycolytic metabolism while knockdown inhibited keratinocyte proliferation and glycolysis. Mice lacking PKM2 specifically in keratinocytes, pharmacological inhibition of PKM2 or glycolysis inhibited keratinocyte proliferation and showed obvious remission in an imiquimod-induced psoriatic mouse model. Moreover, the inhibitor of the EGF-receptor blocked EGF-stimulated PKM2 expression and glycolysis in keratinocytes. We identify PKM2 as an upregulated gene in psoriasis. PKM2 is essential in keratinocyte over-proliferation and may represent a therapeutic target for psoriasis.

## Introduction

Psoriasis vulgaris is a chronic inflammatory dermatological disease, which is characterized by hyper-proliferation and parakeratosis in epidermis, telangiectasia, and inflammation in the dermis (Lowes et al., 2014; Hwang et al., 2017; Zhang et al., 2020). Many studies have focused on the effects of inflammation on psoriasis (Albanesi et al., 2018; Nadeem et al., 2018; Al-Harbi et al., 2020). T cells are considered to be prominent players in psoriasis. They can release various cytokines, such as IL-17A, IL-22, IFN-*γ*, and TNF-*α*, which affect the proliferation/differentiation of keratinocytes in multiple ways (Lowes et al., 2008; Ogawa et al., 2018). Bruton’s tyrosine kinase and interleukin-2-inducible T-cell kinase have the potential to become new therapeutic targets in treating psoriasis inflammation (Nadeem et al., 2020a; Nadeem et al., 2020b). Proliferating cells use glycolysis with reduced mitochondrial oxidative phosphorylation for glucose metabolism, which provides cells with metabolic intermediates used in the duplication of cellular biomass during proliferation (Lunt and Vander, 2011). The psoriatic epidermis is consists of an over-proliferative keratinocytes layer, while keratinocytes are characterized by high levels of glucose metabolism and lactate production (Cruickshank and Trotter, 1956). We postulated that glycolysis might be implicated in psoriasis.

Pyruvate kinase (PK) catalyzes the final step in glycolysis, converting phosphoenolpyruvate to pyruvate while phosphorylating ADP to produce ATP (Vander et al., 2009). In mammalian cells, there are four pyruvate kinase isoforms: the PKL and PKR are expressed in liver and red blood cells, respectively; the PKM1 is expressed in most adult tissues; and the PKM2 is the primary isoform in embryonic tissues and proliferating cells, which is a splice variant of PKM1 (Noguchi et al., 1986; Christofk et al., 2008). PKM2 has been identified as a potential molecular target for disrupting glucose metabolism in cancer cells (Keller et al., 2012). However, whether PKM2 regulates energy metabolism and proliferation in psoriatic keratinocytes remains unknown. Previous studies highlight the importance of EGFR and ERK1/2 signal pathways in the essential functions of PKM2 in cell proliferation and tumorigenesis (Yang et al., 2011; Yang et al., 2012). As both epidermal growth factor receptor (EGFR) activation and ERK1/2 signal pathway are instrumental in psoriasis (Krueger et al., 1990; Johansen et al., 2005), we examined whether EGFR and ERK1/2 activation regulate PKM2 functions in keratinocytes.

Therefore, the key objective of the present study was to investigate the role of PKM2 on psoriatic keratinocytes metabolism and proliferation in both keratinocytes and a mouse model of imiquimod-induced psoriasis. We report here that psoriatic keratinocytes express exclusively the embryonic M2 isoform of pyruvate kinase. We demonstrate that PKM2 is required for glycolysis and proliferation of epidermal keratinocytes. Deletion or inhibition of PKM2 remarkably reduced pathology in an *in vivo* mouse model of psoriasis.

## Materials and Methods

### Materials

DMEM, fetal bovine serum, penicillin, and streptomycin were purchased from GIBCO (GIBCO-BRL, Grand Island, NY, United States). PKM2 shRNA lentivirus and Flag-tagged human PKM2 lentivirus vector were purchased from Oobio (Oobio, Shanghai, China). Puromycin, Hoechst 33342, 2-deoxy-d-glucose, and DMSO were purchased from Sigma-Aldrich (St. Louis, MO). AG-1478 was purchased from MedChemExpress (Monmouth Junction, NJ, United States). EGF was purchased from PeproTech Inc. (Rocky Hill, NJ, United States). IMQ was purchased from 3 M Health Care Limited (United Kingdom). PKM1, PKM2, GAPDH, Flag, p-ERK 1/2, ERK 1/2 antibodies were purchased from Cell Signaling Technology (Beverly, MA). Shikonin was purchased from Shanghai Yuanye Bio-Technology Co., Ltd. (Shanghai, PR China).

### Cell Culture and Treatments

The immortalized human keratinocyte cell line HaCaT cell (maximum passage of 15) and the primary normal human epidermal keratinocytes (NHEK, maximum passage of 5) were all widely used in mechanistic and pharmacological studies of potential skin drugs. HaCaT cells differ from primary keratinocytes in several pathophysiological processes (Pastore et al., 2011). To fully understand the role of PKM2 in keratinocytes, both two human keratinocytes were used. Primary normal human epidermal keratinocytes (NHEK) (Promocell, Heidelberg, Germany) were cultured in keratinocyte growth medium 2. Cells were seeded on collagen-coated dishes and kept constantly sub-confluent to avoid triggering of differentiation. HaCaT cells (oobio, Shanghai, China) were grown in DMEM (GIBCO, United States), and supplemented with 10% fetal bovine serum (GIBCO), 100 U/ml penicillin, and 0.1 mg/ml streptomycin (GIBCO). Cells were cultured at 37°C in a humidified atmosphere of 5% CO_2_. PKM2 expression was knocked down using short hairpin RNA (shRNA). The human PKM2 silencing sequence (PKM2 shRNA 5′-CAG​AGA​AGG​TCT​TCC​TTG​CTC​AGA​A-3′) was subcloned into the pDKD-CMV-eGFP-U6-shRNA adenovirus vector (Oobio, Shanghai, China). The empty vector was used as a negative control. PKM2 expression was over-expressed using Flag-tagged human PKM2, which was cloned into the lentivirus vector pLenti-CMV-EGFP-P2A-MCS-3FLAG (Oobio). To infect cells, multiplicity infection (MOI) of 20 lentiviral particles with 5 μg/ml polybrene were added into the medium, 24 h later the medium was changed and cells were cultured for an additional 72 h. Cells were selected in 2 μg/ml puromycin for 1 week.

### Animals

BALB/c mice (8 weeks old, 20–25 g, female, SPF grade) were supplied by the Shanghai Slac Laboratory Animal CO., LTD. (Slac, Shanghai, China). PKM2^flox/flox^ mice with C57BL/6 genetic background (B6; 129S-Pkm^tm1.1Mgvh^/J) were purchased from The Jackson Laboratories (Bar Harbor, ME). K14-cre mice were generated by Cyagen (Cyagen Biosciences, Suzhou, China). PKM2^flox/flox^ mice were bred with K14-cre mice to generate PKM2^flox/flox^ K14-cre mice. All mice were genotyped by PCR analysis. They were maintained on an automated time cycle of 12-h light/dark at 20°C, with standard food pellets (Shilin, Shanghai, China) and water available ad libitum.

### Imiquimod-Induced Psoriatic Mouse Model

The imiquimod-induced psoriatic mouse model was induced as described previously (van der Fits et al., 2009; Flutter and Nestle, 2013). C57BL/6 mice and BALB/c mice were the two most commonly studied strains in the imiquimod-induced psoriasis model (Flutter and Nestle, 2013; Swindell et al., 2017). Female mice were used because females are generally more sensitive to imiquimod than males (Swindell et al., 2017). Mice dorsal skin was treated daily for 5 consecutive days with 62.5 mg imiquimod cream containing 5% IMQ (3M Health Care Limited, United Kingdom). Vaseline was used as a control. For 2-DG or Shikonin treatment, mice were intraperitoneally injected with 2-DG (500 mg/kg in saline, daily for 5 days) or Shikonin (25 mg/kg in saline/1% DMSO. daily for 5 days). Control mice were injected with 1% DMSO in saline. On day 5, full-thickness skin biopsies of the treated area were collected with an 8-mm biopsy puncher. The skin was either snap frozen in liquid N_2_ for protein lysates preparation, or fixed in paraformaldehyde for histopathological analysis. Mouse serum was collected as described previously (Yang et al., 2013). Average epidermal thickness was quantified by a researcher blind to the experimental groups.

### Human Skin Samples

Human skin biopsies of psoriatic lesions from 10 patients with moderate—to severe psoriasis (defined by a psoriasis area and severity index [PASI] score ≥12 or a total body surface area involvement ≥10%). None of the patients had received any psoriasis treatment except for emollient for at least 3 weeks before the study. Skin samples were also taken from healthy volunteers with no personal or family history of psoriasis (*n* = 10, control group). Specimens were acquired at Shanghai Dermatology Hospital between May of 2017 and Dec of 2017 (Supplementary Table S1).

### Gene Expression Profiling Data Analysis

Gene expression data of psoriatic skin lesions was extracted from the GEO database. PKM gene expression level for each sample was quantified as normalized Reads Per Kilobase of exon model per Million mapped reads (nRPKM), defined as the number of reads aligning to the PKM gene/(total number of uniquely mapped reads for each sample normalized by size factor x PKM gene length). Expression summary values for all probe sets were calculated using the RMA algorithm as implemented in the affy package from Bioconductor (Gautier et al., 2004).

### Detection of Cell Proliferation, Glucose Consumption, Lactate Production, and ATP

Cells (2,000 per well) were seeded into 96-well plates in 100 μL medium and incubated for 96 h. Then, the number of cells was measured by Cell Counting Kit-8 (CCK-8) (Dojindo Laboratories, Kumamoto, Japan) at the indicated time point. Data acquisition was performed on SpectraMax M2e from Molecular Devices (San Jose, CA, United States). For detection of glucose, lactate concentration, cells were seeded in 96-well plates at appropriate concentrations. When cell confluence was 80–90%, the culture medium was replaced by 1 mM glucose growth medium. After different treatments, the supernatant of the culture medium was collected for measurement of glucose and lactate concentrations. The levels of glucose were determined using the Glucose Assay Kit (Sigma-Aldrich) and the levels of lactate were determined using the Lactate Assay Kit (Jianchen, Nanjing, China) under microplate reader (Molecular Devices) according to their respective manufacturer’s protocols. At the same time, the number of cells in each well was counted. Glucose consumption and lactate production were normalized to cell number, respectively. Intracellular ATP levels were determined by the CellTiter-Glo Luminescent Cell Viability Assay (Promega) according to the manufacturer’s instructions.

### Immunohistochemistry

Human and mouse skin tissues were fixed in 4% paraformaldehyde for 24 h and embedded in paraffin. Hematoxylin and eosin (HE) was used to stain 5-μm-thick paraffinized sections. The following primary antibodies were used overnight at 4°C: PKM1 (1:100, Cell Signaling Technology (CST), Beverly, MA), PKM2 (1:100, CST). The sections were counterstained with hematoxylin. The negative controls were performed in the same manner but without the primary antibody.

### Immunofluorescence Staining

Cells were fixed in fresh paraformaldehyde solution, and then incubated with PKM2 antibody (1:100, CST) and Cytokeratin antibody (1:100, CST) over night at 4°C and second antibodies were then loaded for 2 h at room temperature in the dark. After washing, Hoechst was used for nuclear staining. Stained cells were visualized with an Olympus Microscope IX71 (Olympus, Tokyo, Japan). Tissues from six mice per group were used for immunofluorescence staining.

Freshly isolated mouse skin specimens were fixed in 4% paraformaldehyde for 24 h and embedded in paraffin. Nonspecific binding was blocked by treatment with normal goat serum and 0.3% Triton X-100. The tissues were next incubated for 24 h at 4°C with primary rat monoclonal antibodies against PKM2 (1:100, CST) and mouse polyclonal antibodies against Keratin 14 (1:100, Servicebio, Wuhan, China) diluted in primary antibody dilution buffer containing 0.3% Triton X-100. After washing with PBS, immunoreactivity was detected by incubation with FITC with goat anti-rabbit IgG (Servicebio, Wuhan, China) and Cy3 with goat anti-rat IgG (Servicebio, Wuhan, China) diluted in PBS containing 0.5% Triton X-100 for 2 h. An Olympus Microscope IX71 (Olympus, Tokyo, Japan) was used to examine the specimens.

### Western Blot Analysis

Cells or tissues were lysed and the protein concentrations were measured as we described previously (Liu et al., 2017). Briefly, 20 µg of reduced and heat-denatured protein were electrophoresed through 10% sodium dodecyl sulfate (SDS)-polyacrylamide protein gels (prepared in-house) before transfer to NC (Millipore, Australia) membranes. The blotted wet membrane was blocked for 2 h at room temperature with 5% nonfat dried milk. The following primary antibodies were used overnight at 4°C: PKM1 (1:1,000, CST #7067), PKM2 (1:1,000, CST #4053), GAPDH (1:1,000, CST #5174), Flag (1:1,000, CST #14793), p-ERK 1/2 (1:500, CST #4370), ERK 1/2 (1:1,000, CST #9102). After the second antibodies (1:5,000, CST #7074) incubation, luminescence signals on the membrane were detected with Immobilon Western HRP Substrate (Millipore, Darmstadt, Germany) and blots were imaged by an Amersham Imager 600 (GE Healthcare Bio-Sciences AB).

### Ethical Approval and Consent to Participate

All animal procedures used in this study were approved by the Institutional Animal Care and Use Committee of the Second Military Medical University (No. 20170012) and the ARRIVE guidelines. Shanghai Dermatology Hospital review board approval and written informed. The imiquimod-induced psoriatic mouse model has consent was obtained from each outpatient and normal subjects were entered into this study according to the Declaration of Helsinki (No. 20170032).

### Data and Statistical Analyses

Data and statistical analysis comply with the recommendation of experimental design and analysis in pharmacology (Curtis et al., 2018). For all experiments, the number of observations (group size) is provided in the Figure legends, with a minimum of 10 independent observations performed in patient samples. Statistical analysis was undertaken only for studies where each group size was at least *n* = 5. Any data with a sample size of *n* < 5 are indicated as exploratory observations. All cell experiments were performed in triplicates and repeated at least twice. Data analysis and data presentation from these experiments used the single values obtained from the mean of the technical replicates. The declared group size is the number of independent values, and that statistical analysis was done using these independent values. The mice were randomly assigned. All data collection and analysis were performed in a blinded manner. One experimenter carried out the drug administration; the other carried out the data collection and analysis. The data are expressed as the mean ± SEM and analyzed for statistical significance using GraphPad Prism 5.0.1 (Supplementary Table S2, GraphPad Software, La Jolla, CA, United States, RRID: SCR_002798). One-way ANOVA was used to detected statistical significance among group means and Bonferroni post-hoc analysis was used to compare specific groups when ANOVA showed significant differences. *Post hoc* analysis was only performed when the *F* value was greater than *F* critical value, indicating that there was no variance in homogeneity. Statistical significance was set at *p* < 0.05. * indicates statistical significance in all Figures. Outliers were excluded in data analysis and presentation, where indicated. An outlier is predefined when an individual data point is 2 SDs from the mean.

## Results

### PKM2 Expression is Elevated in the Skin of Patients With Psoriasis

**FIGURE 1:**
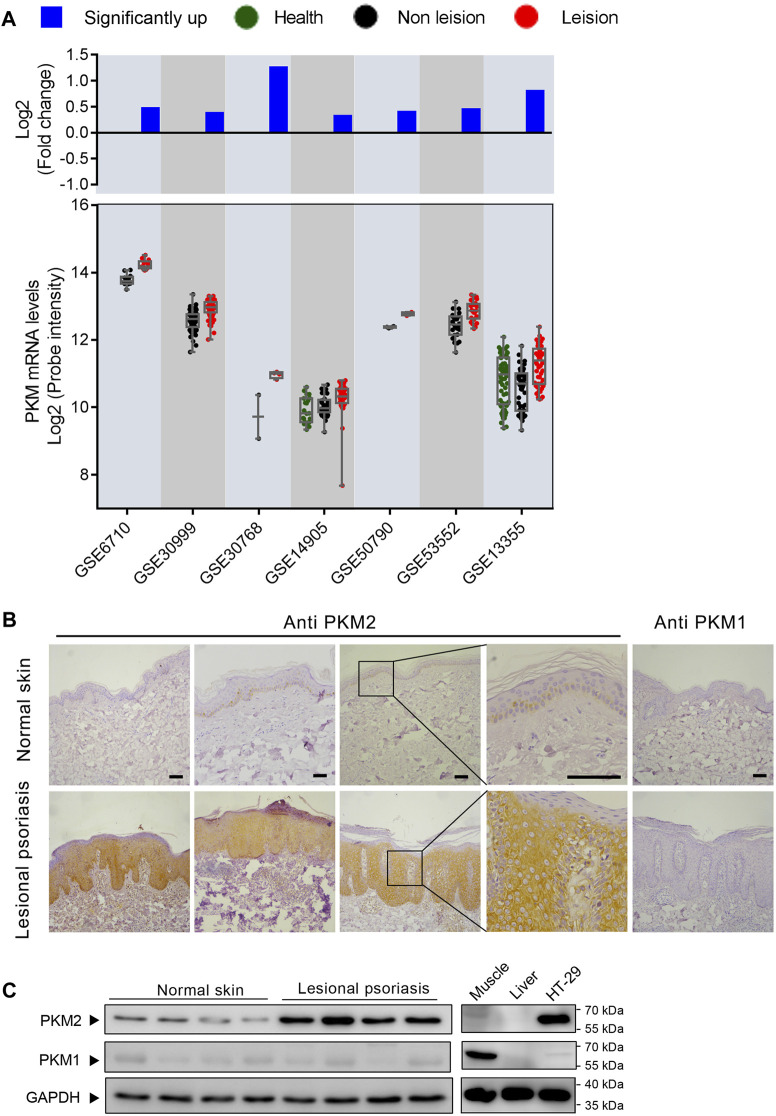
M2 isoform of pyruvate kinase (PKM2) expression is elevated in human psoriatic skin. **(A)** *PKM* is frequently upregulated in human psoriasis lesional skin. Scatterplot showing *PKM* mRNA levels from psoriasis lesional skin and their corresponding normal tissues from the GEO datasets. The fold change between mean psoriasis lesional and mean normal skin expression is shown in the bar plot on a log 2-transformed scale. **(B)** Immunohistochemical staining of serial sections of human skin tissues with antibodies against PKM2 and PKM1. Scale bar: 100 μm. **(C)** Protein levels of PKM2 and PKM1 in the lesions of psoriasis patients and healthy controls. HT-29 is a colorectal adenocarcinoma cell line; Mouse muscle lysate was included as a control for PKM1 antibody staining; Mouse liver lysate was included as a negative control for PKM1 and PKM2 antibody staining. GAPDH was used as loading control.

Our preliminary search using public GEO databases indicated *PKM* expression is elevated in human psoriasis lesional skin (Figure 1A). Notably, seven GEO expression series used in this analysis contained different sample groups including health, psoriasis leision, and non leision. Using this database, our statistical analysis showed that psoriasis leision samples contained a higher level of *PKM* mRNA as compared to health or non leision samples (Figure 1A). Immunohistochemical analysis of tissue specimens with PKM1and PKM2-specific antibodies revealed abundant PKM2 protein in the epidermal layer of skin tissue specimens from different psoriasis patients, whereas very little PKM2 protein was detected in all 10 non-psoriasis control specimens (Figures 1B; Supplementary Figure S7). As shown in Figure 1C, the levels of PKM2 were over-expressed in the epidermis of psoriatic lesions than that in normal skin. PKM1 was absent or present in a very low amount from normal skin by both immunohistochemistry (Figure 1B) and Western blotting (Figure 1C).

### Increased Expression of PKM2 in Skin Lesions From Imiquimod-Treated Mice

**FIGURE 2:**
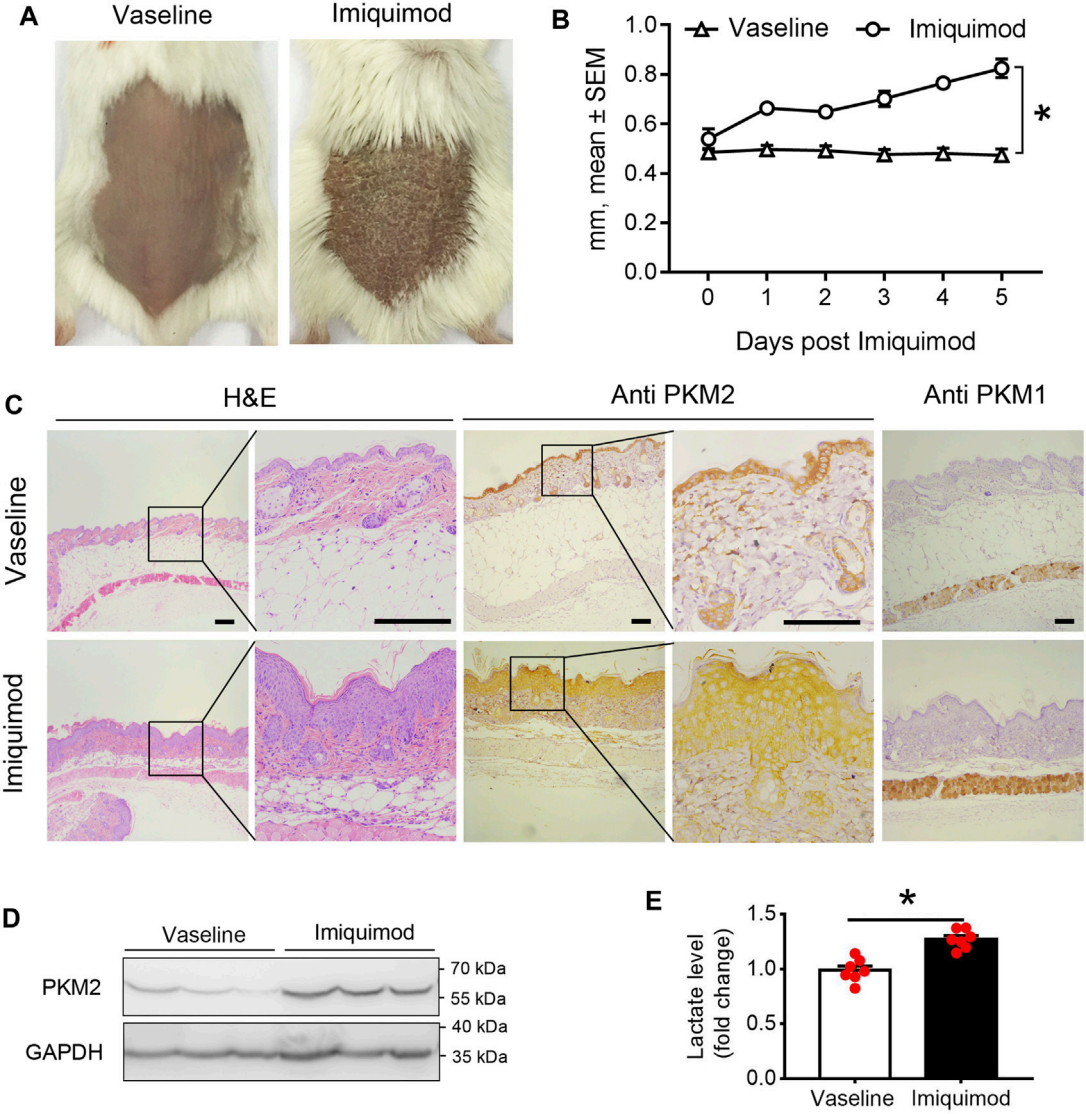
PKM2 expression is elevated in imiquimod (IMQ)-induced psoriatic mouse skin. IMQ or control cream (vaseline) was applied daily to the shaved backs for female BALB/c mice. **(A)** Phenotypical presentation of mouse back skin after 5 days of control cream (left panel) or IMQ treatment (right panel). **(B)** The skin thickness was measured on the days indicated. Significant differences are indicated, *n* = 7 per group (mean ± SEM), *p* < 0.01. **(C)** Light microscopy examination of skin sections stained with H&E, or with Abs against the PKM2 and PKM1. Scale bars, 100 μM. H&E, haematoxylin and eosin. **(D)** Representative immunoblotting of PKM2 from skin samples of control cream or IMQ treated mice on day 5. **(E)** Serum lactate level is elevated in IMQ treatment mice compared to control cream on day 5 (*n* = 7, *p* < 0.001). GAPDH was used as loading control. IMQ, Imiquimod.

We assessed PKM2 expression in the skin from the backs of mice treated with imiquimod (IMQ) for five consecutive days. As shown in Figures 2A,B; Supplementary Figure S1, we found that IMQ-treated skin developed signs of erythema, scaling, and thickening over the course of treatment. Within the psoriasis-like skin lesions, PKM2 protein expression was significantly increased in hyper-proliferative keratinocytes (Figures 2C; Supplementary Figure S8), and Western blotting revealed a corresponding elevation of PKM2 expression (Figure 2D). PKM1 was absent or present in a very low amount in keratinocytes and strongly expressed in muscle tissues (Figure 1C, right panels). In addition, we examined the mice’s serum lactate levels after IMQ treatment. The lactate production (a glycolytic end product) was significantly elevated in IMQ-treated mice serum (Figure 2E).

### PKM2 Is Essential for Keratinocyte Cell Glucose Metabolism and Proliferation

**FIGURE 3:**
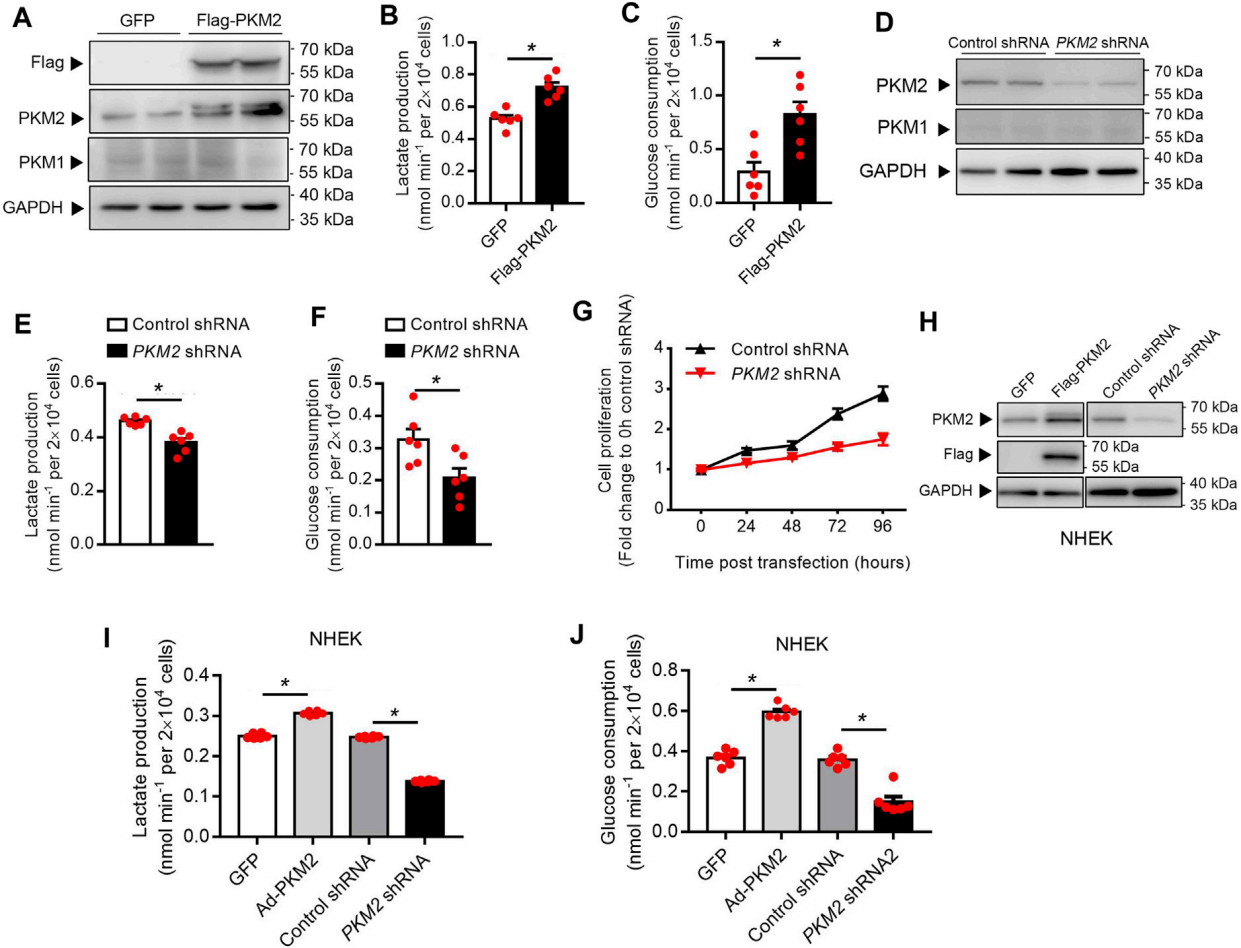
PKM2 play a critical role in the keratinocyte glucose metabolism and proliferation. **(A)** Immunoblotting of HaCaT cell stably expressing Flag-PKM2. Cells were infected with lentivirus containing the empty vector (GFP) or Flag-PKM2 (MOI = 40). The cells were then selected in puromycin (2 μg/ml) for 1 week. Total cell extracts were immunoblotted with antibodies for PKM1, PKM2, Flag, and GAPDH. **(B, C)** Lactate production and glucose consumption were measured in HaCaT-GFP and HaCaT-Flag-PKM2 cells. **(D)** Efficiency of *PKM2* shRNA knockdown. Immunoblot analyses indicate ∼70% reduction of PKM2 in *PKM2* shRNA-expressing cells compared with control shRNA. **(E, F)** Lactate production and glucose consumption were measured in HaCaT-control shRNA and HaCaT-*PKM2* shRNA cells. **(G)** Proliferation curves of the PKM2 knockdown and control cells. **(H)** Immunoblotting of NHEK treated with Flag-PKM2 lentivirus or *PKM2* shRNA adenovirus. **(I, J)** Lactate production and glucose consumption were measured in NHEK. NHEK were transfected with Flag-PKM2 lentiviruses or PKM2 shRNA adenovirus. NHEK: normal human epidermal keratinocytes. Data are means ± SEM (*n* = 6). GAPDH was used as loading control.

To examine whether PKM2 expression enhances keratinocyte aerobic glycolysis, a lentivirus vector expressing human *PKM2* HaCaT cells was successfully established (Figure 3A). Over-expression of PKM2 in HaCaT cells increased glucose consumption and lactate production compared with control cells (Figures 3B,C). Besides, to interrogate the effect of PKM2 knockdown on keratinocyte cell glucose metabolism and proliferation, Untreated HaCaT cells were transfected with PKM2 shRNA or control shRNA (Figure 3D). Data shows that glucose uptake and lactate production were significantly decreased in PKM2 shRNA treated cells as compared to control shRNA treated cells (Figures 3E,F). The proliferation of the PKM2 shRNA cells was also significantly decreased compared to the control cells (Figure 3G). This was further confirmed in normal human epidermal keratinocytes (NHEK, Figures 3H–J). Altogether, these results suggested a key role of PKM2 in modulating glucose metabolism and proliferation.

### PKM2 Inhibitor Reduces Keratinocyte Cell Glycolysis and Proliferation

**FIGURE 4:**
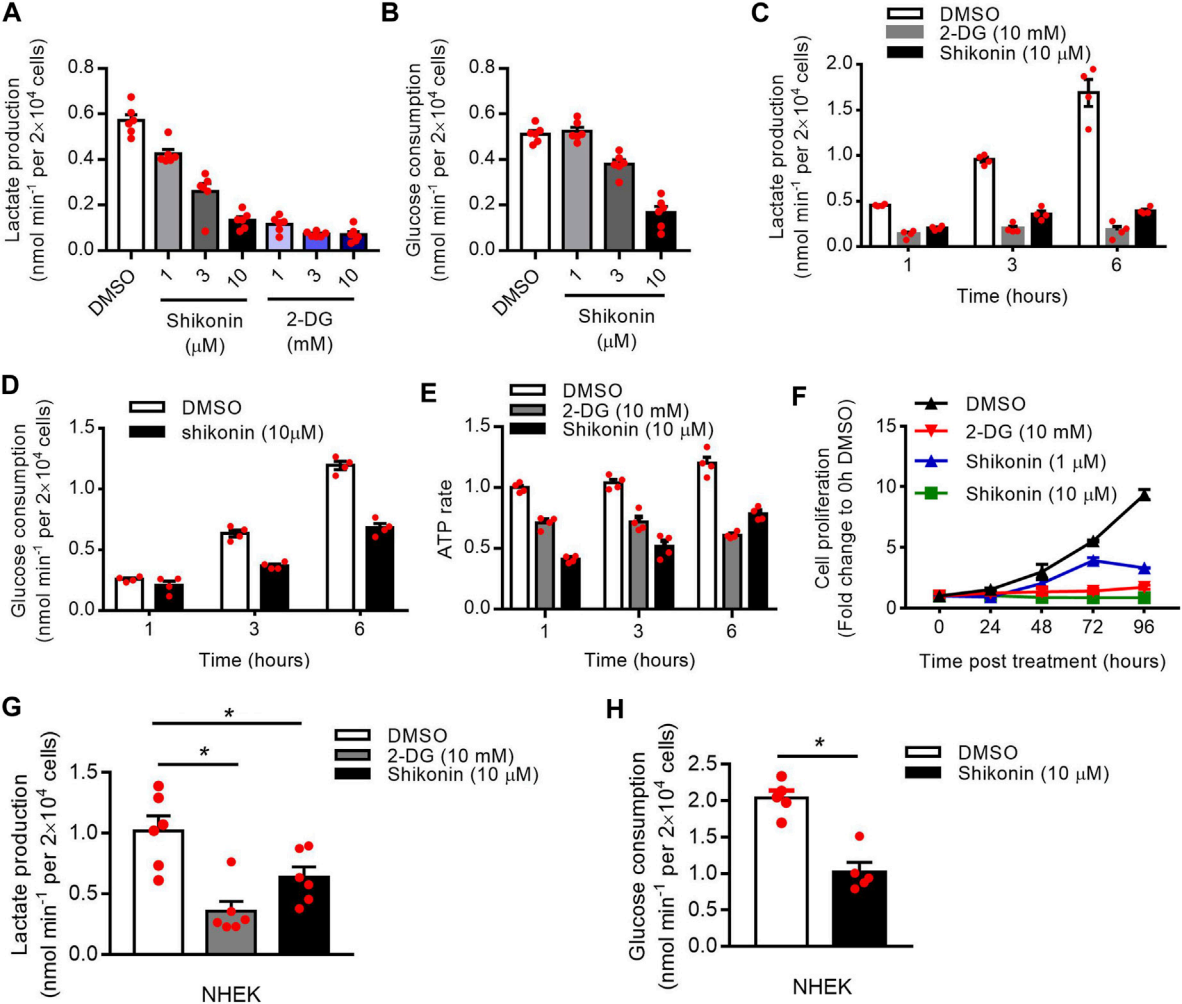
Effects of shikonin and 2-DG on the keratinocyte glucose metabolism and proliferation. **(A)** The lactate production in the supernatants of HaCaT cells after stimulated with different concentrations of shikonin or 2-DG for 1 h was determined using a Lactate Assay kit. **(B)** HaCaT cells were stimulated with different concentrations of shikonin for 1 h and the glucose consumption in the supernatants was analyzed. **(C, D)** The lactate production and glucose consumption in the supernatants of HaCaT cells after stimulated with shikonin or 2-DG for indicated times were determined. **(E)** Intracellular ATP levels following treatment with shikonin (10 μM) or 2-DG (10 mM). ATP concentration was quantified using a luciferase based ATP assay. **(F)** The effects of shikonin and 2-DG on cell proliferation of HaCaT cells were determined by CCK-8 assay. **(G)** Effects of shikonin and 2-DG on lactate production of NHEK cells. **(H)** The glucose consumption in NHEK cells following shikonin treatment. The final DMSO concentration was 0.1% DMSO (v/v). 2-DG, 2-deoxy-d-glucose; NHEK, normal human epidermal keratinocytes. Data are means ± SEM (*n* = 6).

Shikonin is a small-molecule natural product that inhibits the activity of PKM2 (Chen et al., 2011). 2-deoxy-d-glucose (2-DG) is an analog of glucose that inhibits glycolysis (Mohanti et al., 1996). To investigate the function of PKM2 in glycolysis and proliferation, we first treated HaCaT cells with shikonin and 2-DG. Our results showed a dose-dependent decrease in the production of lactate (Figures 4A,C) and glucose uptake (Figures 4B,D) in the supernatants of HaCaT cells after Shikonin or 2-DG (only in lactate production) treatment. Meanwhile, Shikonin or 2-DG-stimulated HaCaT cells also showed a significant decrease in ATP synthesis rates (Figure 4E) and cell proliferation (Figure 4F; Supplementary Figure S2). The influence of shikonin on cell cycle progression is studied using a flow cytometer to analyze cell percentages in each cycle phase: G1/G0, S, and G2/M, and results are shown in Supplementary Figure S3. After 24 h culture, increased percentages of S and G2/M phase can be observed in cells treated with 10 μM shikonin compared with the control group (Supplementary Figure S3). Using NHEK, we also showed that Shikonin or 2-DG (only in lactate production) induced a significant decrease in the production of lactate (Figure 4G) or glucose uptake (Figure 4H). To determine the effect of PKM2 activation on keratinocyte proliferation, PKM2 selective agonist DASA-58 was used. We show that DASA-58 inhibited the proliferation of Hacat cells in a dose-dependent manner (Supplementary Figure S4).

### IMQ-Induced Psoriasiform Dermatitis is Alleviated in Conditional PKM2 Knockout Mice

**FIGURE 5:**
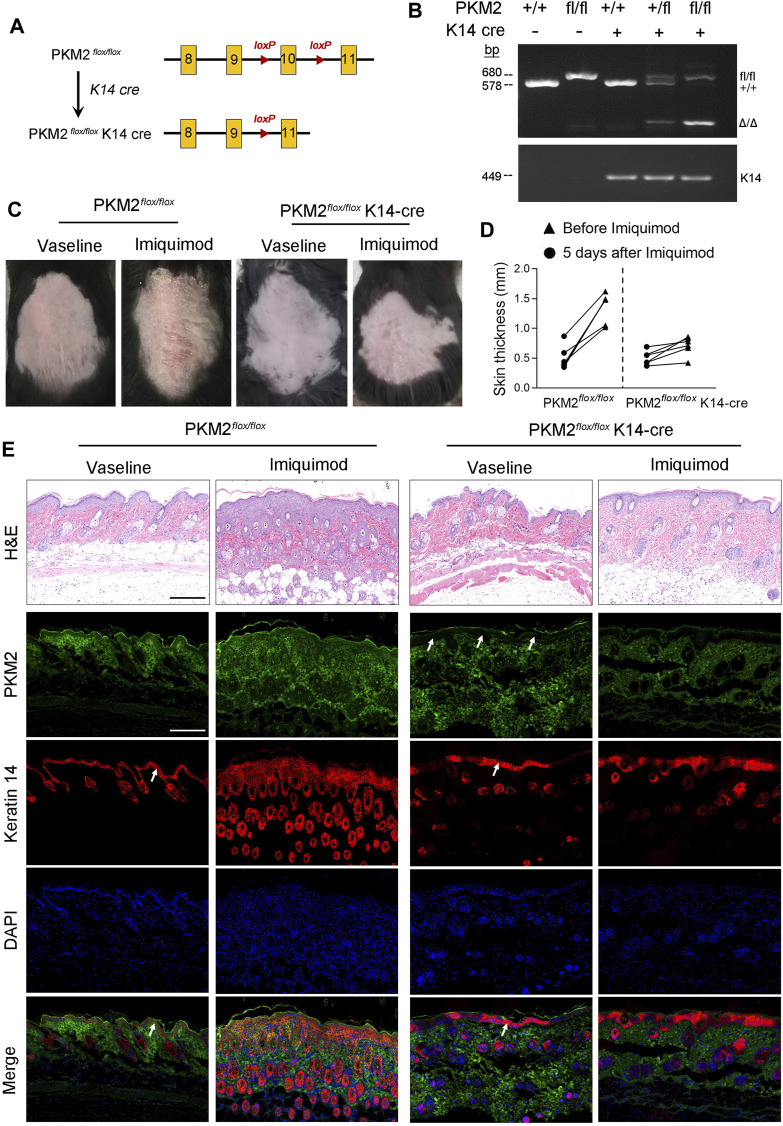
PKM2 deficiency in keratinocytes reduced cutaneous signs and epidermal thickness on the IMQ-induced psoriasis mouse model. **(A)** Breeding strategy to develop PKM2^flox/flox^ K14-Cre mouse. **(B)** PCR showing genotyping of PKM2^flox/flox^ and PKM2^flox/flox^ K14-Cre mice. Band in the upper image indicates the product from PKM2^flox/flox^, and lower image indicates product from K14-Cre. **(C)** Representative images of the dorsal skin after IMQ treatment for 5 days. **(D)** The skin thickness was measured after IMQ treatment for 5 days *n* = 6 per group. **(E)** Representative images of skin sections stained with H&E or the indicated antibodies from mice of the indicated genotypes after IMQ treatment for 5 days. Arrows indicate the epidermis. Nuclei were stained with DAPI (blue). Scale bar, 200 µm.

We generated mice that lack PKM2 selectively in epidermal keratinocytes by crossing PKM2-floxed mice with mice expressing the Cre recombinase under the control of the keratin 14 (K14) promoter (Figure 5A). The genotype of the mice was confirmed by PCR (Figure 5B). At the protein level, the deletion of PKM2 in keratinocytes was verified by immunofluorescence (Figure 5E). As expected, hyperkeratosis and epidermal hyperplasia were present in PKM2^flox/flox^ mice but subjectively reduced in PKM2^flox/flox^ K14-cre mice after 5 days of topical IMQ treatment (Figure 5C). Epidermal hyperplasia was significantly decreased in PKM2^flox/flox^ K14-cre mice vs PKM2^flox/flox^ mice (Figures 5D,E). Supplementary Figure S5 showed expression of PKM1 remained unchanged after IMQ treatment. In addition, PKM2 deficiency in keratinocytes reduced serum lactate levels on the IMQ-induced psoriasis mouse model (Supplementary Figure S6).

### Therapeutic Effect of Shikonin or 2-DG on the IMQ-Induced Psoriasis Mouse Model

**FIGURE 6:**
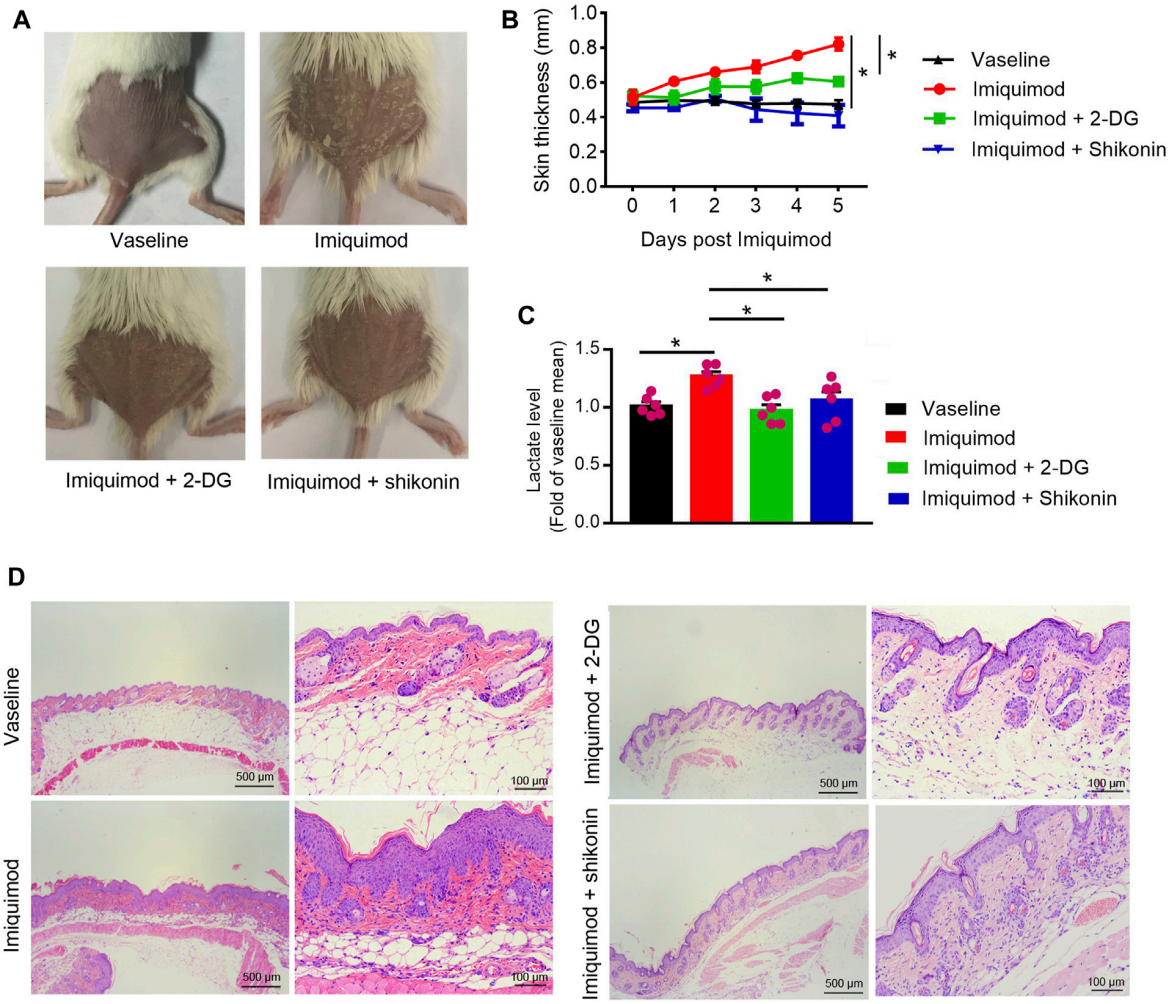
Therapeutic effect of Shikonin or 2-DG on the IMQ-induced psoriasis mouse model. Topical treatment of IMQ cream (5%) for 5 d on the shaved back skins of BALB/C mice. Vaseline was used as a control. Skin samples were collected before euthanasia. **(A)** Pictures of the back skin of the mice before euthanasia. **(B)** The skin thickness was measured on the days indicated. Significant differences are indicated, *n* = 7 per group (mean ± SEM), *p* < 0.01. **(C)** Serum lactate level was measured on day 5 (*n* = 6). **(D)** Pathological analysis with H&E staining of the back skin samples of mice from different treatment groups. IMQ, Imiquimod; 2-DG, 2-deoxy-d-glucose.

The application of IMQ on the shaved back skin of BALB/c mice produced a psoriatic-like lesion, exhibiting signs of erythema, scaling, and thickening. Shikonin or 2-DG treatment significantly attenuated the severity of skin lesions in animals (Figure 6A). The group that received Shikonin or 2-DG treatment showed a significant reduction in skin thickness (Figure 6B). Lactate measurement showed decreased serum lactate levels in the Shikonin or 2-DG treatment IMQ-induced mice, compared with that in the IMQ treatment group (Figure 6C). Histological examination with H&E staining also showed decreased epidermal thickening in the Shikonin or 2-DG treatment group, compared with that in the IMQ group (Figure 6D). These results suggested that PKM2 inhibition may be an important approach for psoriasis treatment.

### EGF Stimulates Keratinocyte PKM2 Expression via the ERK1/2 Pathway

**FIGURE 7:**
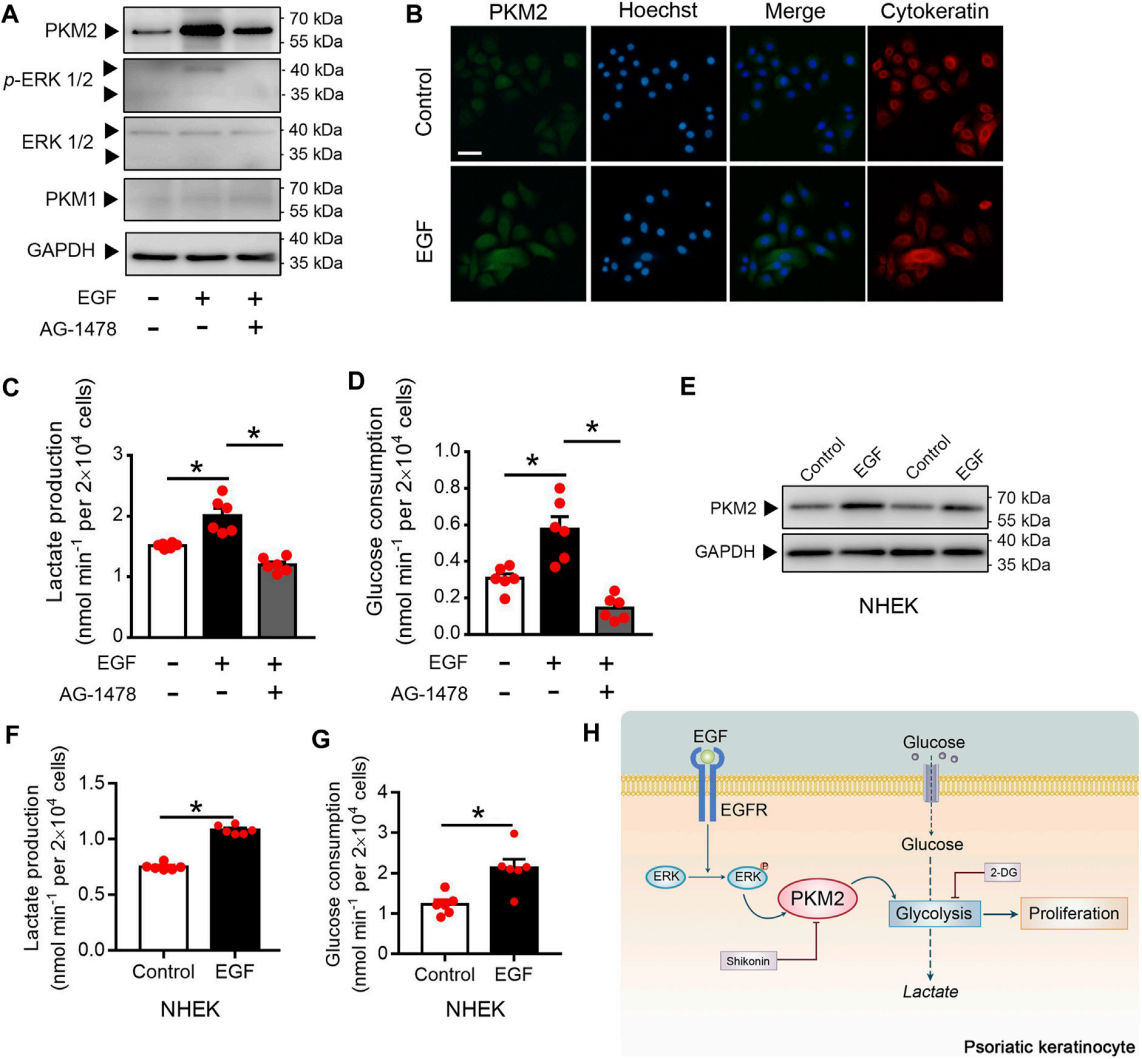
EGF induces the PKM2 up-regulation and glycolysis in the keratinocyte. **(A)** HaCaT cells were pre-treated with or without AG1478 (1 μΜ) for 2 h, and followed incubation of EGF (100 ng/ml) for 24 h. The cell lysates were collected and PKM1, PKM2, ERK, and *p*-ERK were detected by Western blot analysis. **(B)** HaCaT cells were treated with EGF (100 ng/ml) for 24 h. Immunofluorescence analyses were performed with the indicated antibodies. Nuclei were stained with Hoechst 33,258 (blue). Bar = 50 μm. **(C, D)** Cultured HaCaT cells were treated with or without EGF (100 ng/ml) for 24 h in the presence or absence of AG1478 (1 μΜ). Lactate production and glucose consumption were measured in the cell medium. **(E)** NHEK cells were incubated with EGF (100 ng/ml) for 24 h. The cell lysates were collected and PKM2 were detected by Western blot analysis. **(F, G)** Lactate production and glucose consumption in NHEK cells following EGF treatment (100 ng/ml) for 24 h. **(H)** Summary of PKM2-dependent reprogramming of central carbon metabolism and proliferation in psoriatic keratinocyte. GAPDH was used as loading control.

Since EGFR activation is instrumental in psoriasis (Nanney et al., 1986; Flisiak et al., 2014), we examined whether EGFR activation regulates PKM2 expression in the keratinocyte. Immunoblotting analysis showed that EGF treatment increases the expression of PKM2 and the phosphorylation of ERK in the HaCaT cells (Figure 7A). Cells treated with AG-1478 (an EGFR tyrosine kinase inhibitor) exhibited less PKM2 expression and ERK phosphorylation (Figure 7A), indicating that the enhanced PKM2 expression observed was due to EGFR stimulation. The finding that EGF increases expression of PKM2 was further supported by immunofluorescence analysis in HaCaT cells (Figure 7B). Glucose metabolism assay demonstrated that EGF stimulated the production of lactate (Figure 7C) and glucose uptake (Figure 7D) in the HaCaT cells, which was reversed by AG-1478. The effects of EGF on PKM2 expression and glucose metabolism were also evaluated in NHEK cells. EGF treatment stimulated the PKM2 expression (Figure 7E) and reprogrammed the glucose metabolism (Figures 7F,G) in human primary epidermal keratinocytes.

## Discussion

We have uncovered a previously unreported role for the glycolytic protein PKM2 in psoriatic keratinocytes. Collectively, our findings identify a critical role for PKM2-driven metabolic reprogramming in glycolysis and over-proliferation in keratinocyte cells. Inhibition or deletion of PKM2 strongly reduces keratinocyte proliferation in IMQ-induced psoriasis-like skin lesions in this mouse model. Together, these data suggest that blocking PKM2 may represent a new therapeutic strategy in psoriasis (Figure 7H).

Psoriasis is a chronic inflammatory skin disease characterized by epidermal hyperplasia and leukocyte infiltration. Clinical trials have shown therapies targeting IL-12/IL-23 (p40), IL-23 (p19), and IL-17 or its receptor (IL-17RA) to be efficacious (Martin et al., 2013; Sofen et al., 2014). However, several side effects in many patients made these agents not entirely satisfactory, especially when administered over long periods (Yiu et al., 2016). It has been known for some time that the epidermis is a glycolytic tissue (Cruickshank and Trotter, 1956). The systems metabolomics approach revealed that the glycolysis pathway is increased in patients with psoriasis (Kang et al., 2017). Positron emission tomography/Computed Tomography (PET/CT) images also demonstrated cutaneous fludeoxyglucose (FDG) uptake corresponding to clinically apparent psoriatic lesions (Bruna-Muraille et al., 2011). It is well-known that PKM2 participates in glycolysis and provides energy and intermediate products for other biosynthesis in proliferative cells (He et al., 2017). Hao et al. recently reported that sirtuin 2 (Sirt2) deacetylase regulated the protein kinase function of PKM2 in Th17 cell-mediated inflammatory responses in psoriasis (Hao et al., 2021a; Hao et al., 2021b). Even though Th17 cells are important in psoriasis. Our immunohistochemistry and immunofluorescence results show that PKM2 is mainly expressed in keratinocytes of psoriatic skin. Keratinocyte-specific knockout of PKM2 alleviates the progression of psoriasis in mice. These results drive us to believe that keratinocytes PKM2 may play a significant role in psoriasis development.

The oligomers of PKM2 exist in high activity tetramer and low activity dimer forms (Anastasiou et al., 2012). Low catalytic activity PKM2 prefers to prompt glucose-derived carbon to the direction of glycolysis (Christofk et al., 2008), resulting in a diversion of glycolytic metabolites into pathways that make nucleotides, amino acids, and lipids. Paradoxically, Low PK activity is also associated with increased lactate production. Thus, we propose that PKM2, abnormally expressed in psoriasis, is in the low activity dimer forms. Low PK activity would promote keratinocyte proliferation as this would result in a diversion of glycolytic intermediates away from the TCA cycle into biosynthetic pathways.

Shikonin is the active chemical component of Lithospermum erythrorhizon (Zicao), a traditional Chinese medicine to cure psoriasis (Andujar et al., 2013; Wang et al., 2015). However, detailed mechanisms and the targets of shikonin in psoriasis remain unclear. Chen *et al.* demonstrated that shikonin inhibits cell glycolysis and proliferation by targeting PKM2 (Chen et al., 2011). Our results revealed that PKM2 was up-regulated in psoriatic keratinocytes. Inhibition of glycolysis significantly changed IMQ-induced skin pathology. These results suggested that shikonin improves IMQ-induced psoriasis-like skin lesions in mice by inhibiting the PKM2 in keratinocytes.

Furthermore, we investigated the mechanism of PKM2 up-regulation in psoriatic keratinocytes. EGFR, a ubiquitously expressed receptor tyrosine kinase, is important in the control of cell proliferation and survival (Nicholson et al., 2001). EGFR and its ligands, transforming growth factor-alpha (TGF-а) are overexpressed in psoriatic epidermis compared with normal keratinocytes (Powell et al., 1999). Yang et al. demonstrate that EGFR-activated ERK2 binds directly to PKM2 through the ERK2 docking groove and phosphorylates PKM2 at Ser 37 (Yang et al., 2012). In our study, EGF stimulated ERK phosphorylation and PKM2 expression. EGFR inhibitor AG-1478 reversed this effect. These results suggested that EGFR could account for the PKM2 up-regulation in psoriatic keratinocytes. Interestingly, increasing evidence indicates that PKM2 could also regulate gene transcription in the nucleus (Yang et al., 2011; Gao et al., 2012). However, the importance of these non-canonical functions in PKM2-mediated cell proliferation is still under debate (Hsu and Hung, 2018). The role of PKM2’s gene regulation function and related signal transduction pathway in psoriatic keratinocytes is unclear, but worthy of further study.

Over the last four decades, more than 40 unique mouse models for psoriasis have been described (Hawkes et al., 2018). The imiquimod-induced mouse model, which was used in this study, has become a widely used standard to model human psoriasis since its introduction in 2009 and seems to mirror psoriasis in many features (van der Fits et al., 2009). However, the model’s response to anti-psoriatic drugs still needs to be shown. In our study, data in an imiquimod-induced mouse model was consistent with that in clinical specimens. Primary keratinocytes from the human skin experiment also supported the imiquimod-induced mouse model result. Although our *in vitro* and *in vivo* data presented in this paper strongly suggest that PKM2 may be considered as a possible therapeutic target for psoriasis. More psoriatic animal models should be used to test this hypothesis in the future.

In summary, we demonstrated that PKM2 participates in the pathogenesis of psoriasis. PKM2 expression is significantly elevated in human psoriatic skin lesions and IMQ-induced mice skin lesions. Inhibition of PKM2 reduced glycolytic metabolism and inhibited keratinocyte proliferation *in vitro* and *in vivo*. We have thus provided a novel therapeutic approach in which targeting the PKM2 for the specific treatment of psoriasis.

## Data Availability

The datasets presented in this study can be found in online repositories. The names of the repository/repositories and accession number(s) can be found in the article/Supplementary Material.
