# Supplementary material for: Pyruvate Kinase M2 Mediates Glycolysis Contributes to Psoriasis by Promoting Keratinocyte Proliferation
Source: Front Pharmacol. 2021 Oct 18;12:765790. doi: 10.3389/fphar.2021.765790 (PMC8558409; doi:10.3389/fphar.2021.765790)
Supplement: Supplementary file 1 [file DataSheet1.docx]

**Pyruvate kinase M2 mediates glycolysis contributes to psoriasis by promoting keratinocyte proliferation**

Yun-zi Liu^a, c, 1^, Ming-yuan Xu^b, 1^, Xiao-yu Dai^a, 1^, Lang Yan^a, 1^, Lei Li^d^, Rui-zhen Zhu^b^, Li-jun Ren^a^, Ji-qian-zhu Zhang^a^, Xiao-fang Zhang^a^, Jin-feng Li^a^, Yi-jun Tian^a^, Wen-jing Shi^a^, Ye-qiang Liu^b^, Chun-lei Jiang^c^, Jiang-bo Zhu^a, *^, Ji-kuai Chen^a, *^

a. Department of Health Toxicology, Faculty of Naval Medicine, Second Military Medical University, Shanghai, China.

b. Department of Dermatopathology, Shanghai Skin Disease Hospital affiliated to Tongji University, Shanghai b0044c, China.

c. Laboratory of Stress Medicine, Faculty of Psychology and Mental Health, Second Military Medical University, Shanghai, China.

d. The Second Naval Hospital of the Southern Theater of the Chinese People's Liberation Army

***Corresponding Author:**

E-mail: chenjk@smmu.edu.cn (Ji-kuai Chen).

E-mail: jiangbozhu1@163.com (Jiang-bo Zhu)

^1^ Equal first authors.

Yun-zi Liu, Ming-yuan Xu, Xiao-yu Dai, Lang Yan contributed equally to this work.

**Methods**

**Cell-cycle analysis**

Cells were treated with shikonin (0, 1, 3 and 10 μM) for 24 h. After treatments, the cells were collected by trypsinization, washed with ice-cold PBS and fixed in ice-cold 70% methanol by incubating them for 1 h at 4°C. The cells were then centrifuged, suspended in PBS and incubated with RNase for 30 min at 37°C. The cells were then stained with PI for 1 h and analyzed by CytoFLEX S flow cytometer and FlowJo V10 (Flowjo, OH, USA).


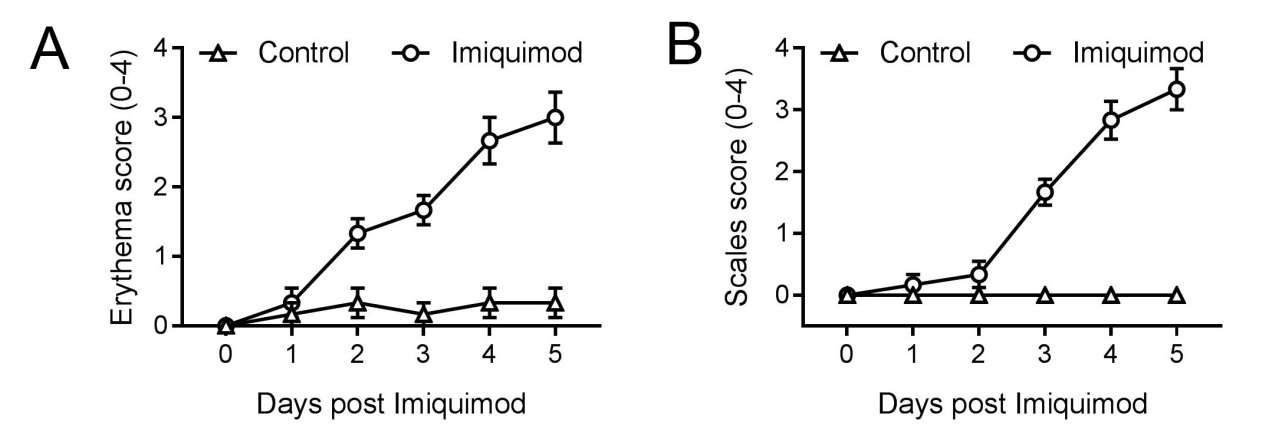
**Figure S1. Imiquimod (IMQ)-induced psoriasis-like skin in mice.** Female BALB/c mice were treated daily with IMQ cream or control cream on the shaved back skin. A and B, Erythema and scaling of the back skin was scored daily on a scale from 0 to 4. Symbols indicate mean score ± SEM of six mice per group.


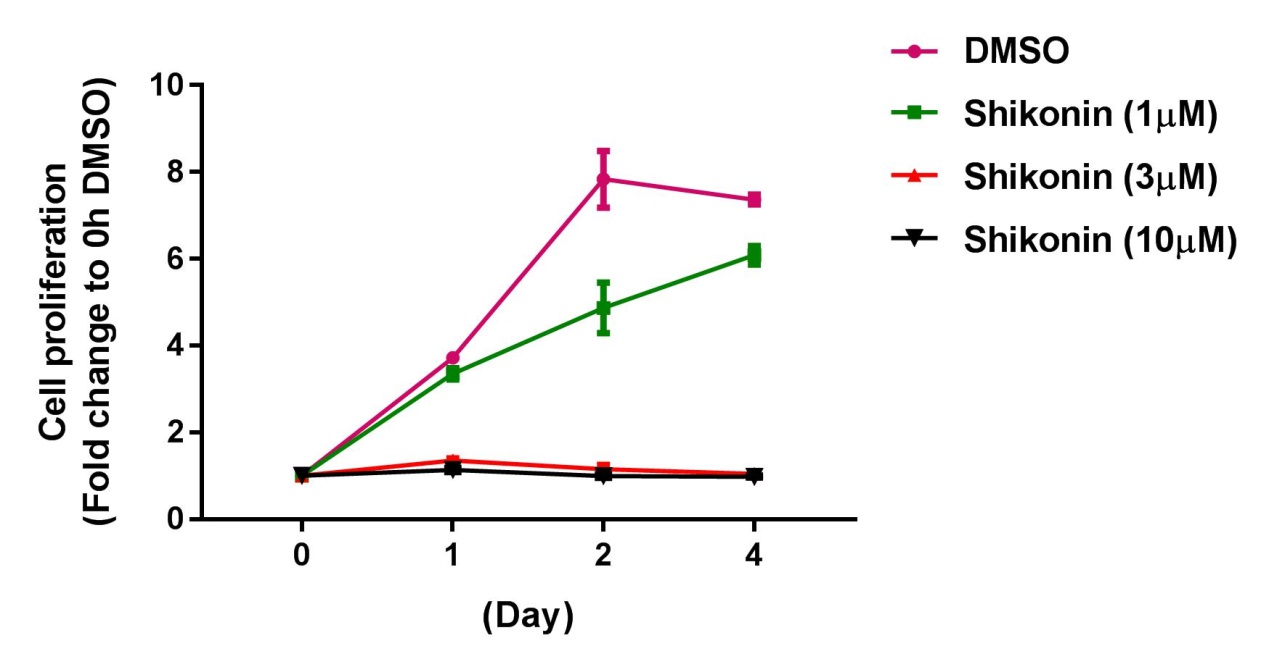


**Figure S2 Effects of shikonin on the keratinocyte proliferation.** Hacat cells were stimulated with different concentrations of shikonin. The cell proliferation of Hacat cells were determined by CCK-8 assay. Data are means ± SEM (n = 6).


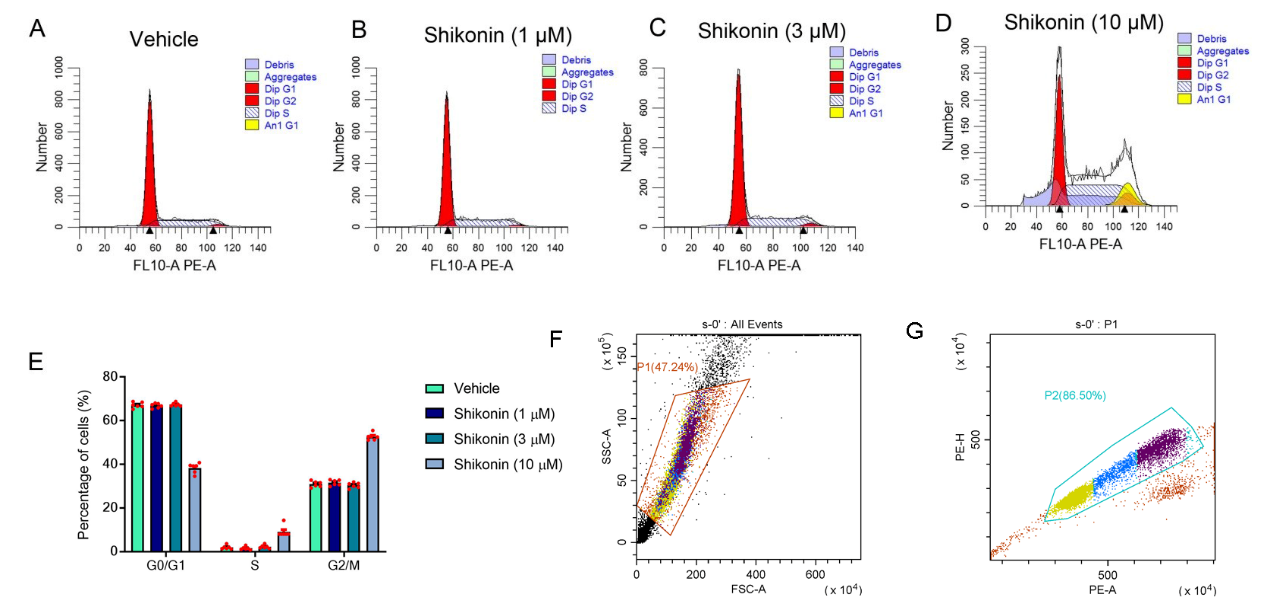


**Figure S3 Effects of shikonin on HaCaT cell cycle progression.** HaCaT cells were treated with 1, 3 or 10 µM shikonin for 24 h. (A) control group, (B) 1 µM shikonin, (C) 3 µM shikonin, (D) 10 µM shikonin, (E) graphic representations of the cell cycle distributions of HaCaT cells. (F) and (G) the complete gating strategy of the flow cytometry analysis. Data are means ± SEM (n = 6). *P<0.05 vs. the control group.
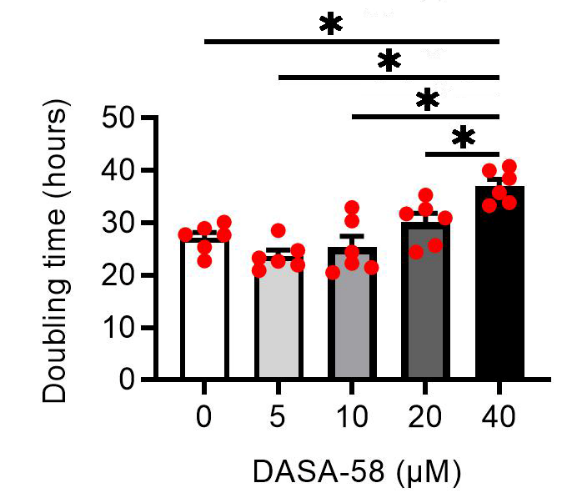


**Figure S4 Effect of PKM2 activators on cell proliferation.** Effects of DASA-58 (used at 5, 10, 20, and 40 μM) on the doubling time of Hacat cells (n = 6, * P < 0.05).


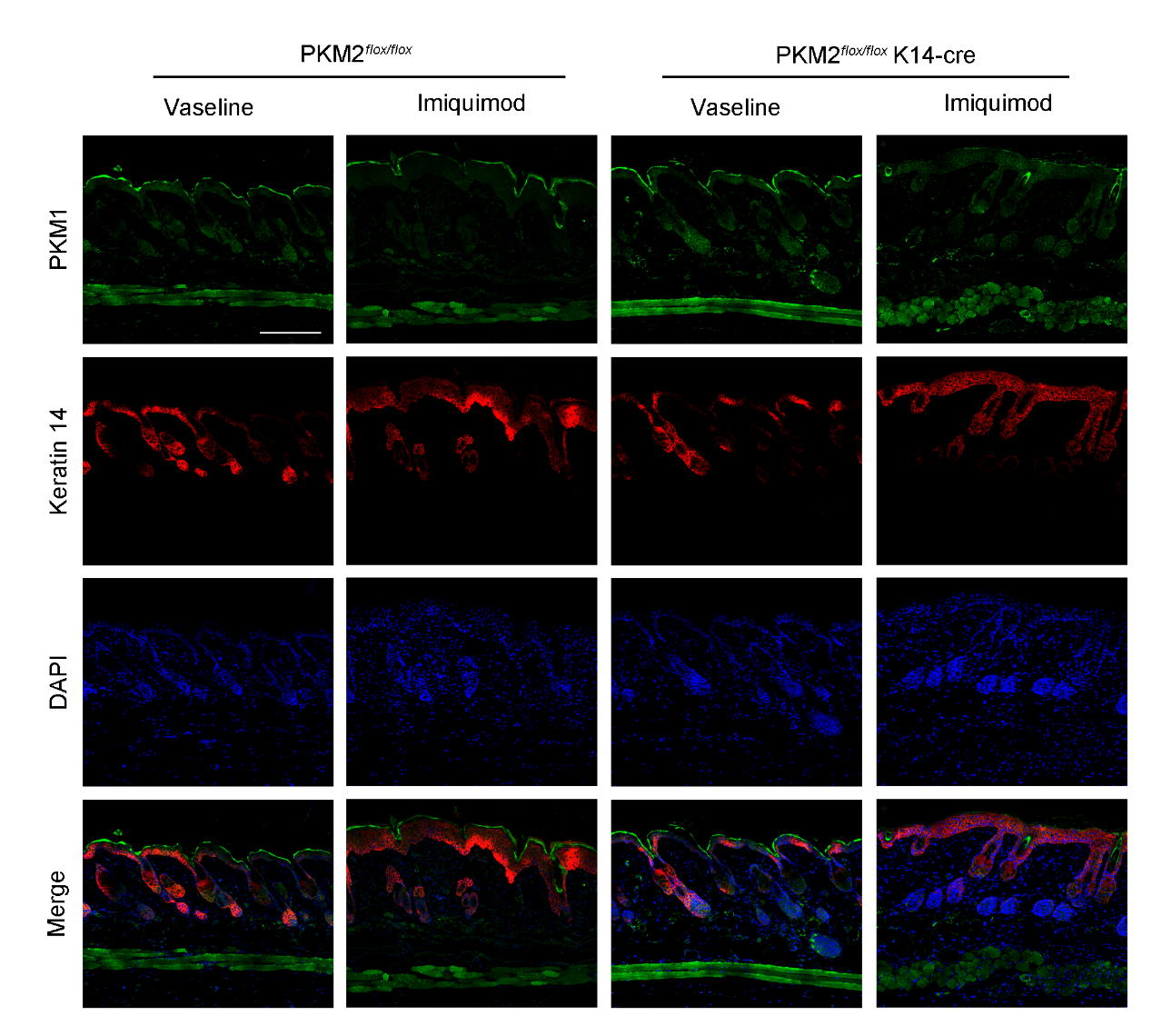


**Figure S5 Immunofluorescence analysis of PKM1 in mice with PKM2 deficiency in keratinocytes on the IMQ-induced psoriasis mouse model.** Representative images of skin sections stained with the indicated antibodies from mice of the indicated genotypes after IMQ treatment for 5 days. Nuclei were stained with DAPI (blue). Scale bar, 200 µm.


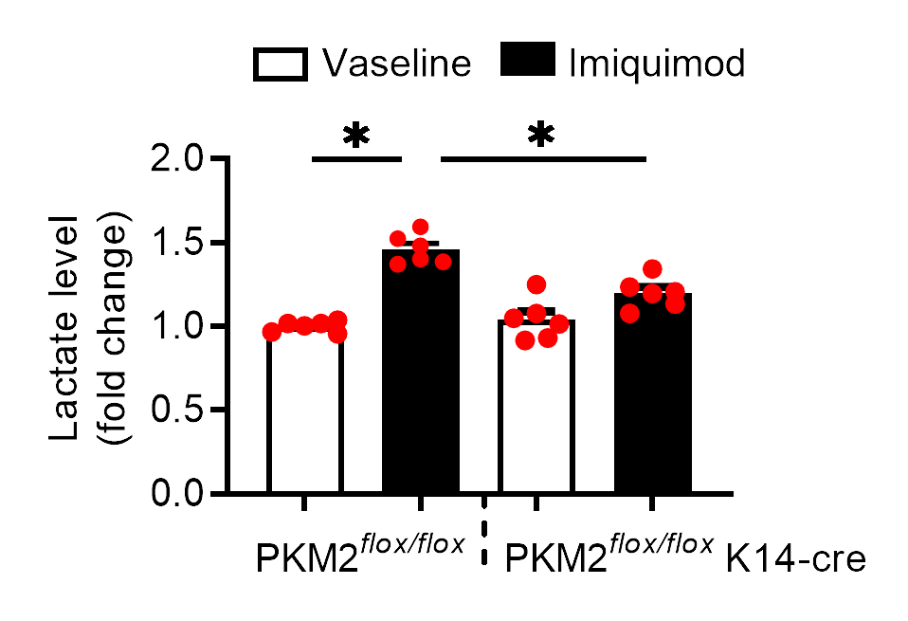


**Figure S6 PKM2 deficiency in keratinocytes reduced serum lactate levels on the IMQ-induced psoriasis mouse model.** Serum lactate level is reduced in IMQ treatment PKM2*^flox/flox^* K14-cre mice compared to PKM2*^flox/flox^* mice on day 5 (n = 6, * P < 0.001).


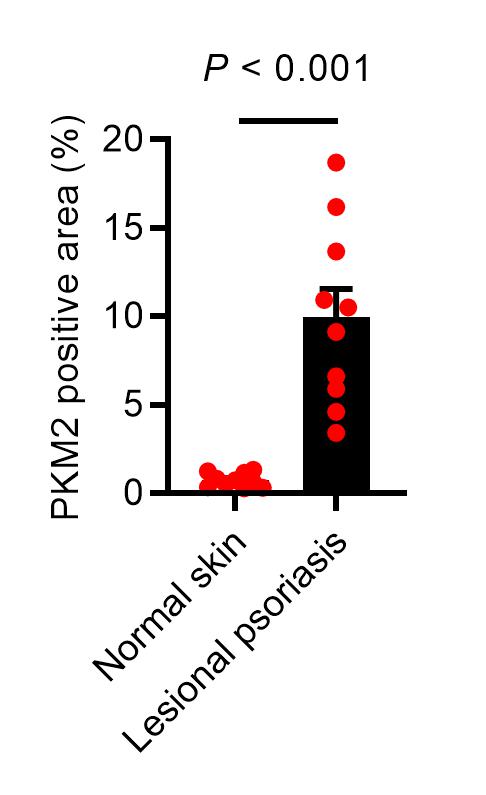


Figure S7 Quantitative results of PKM2 positive staining area percentage by immunohistochemistry in the lesions of psoriasis patients and healthy controls. n=10.


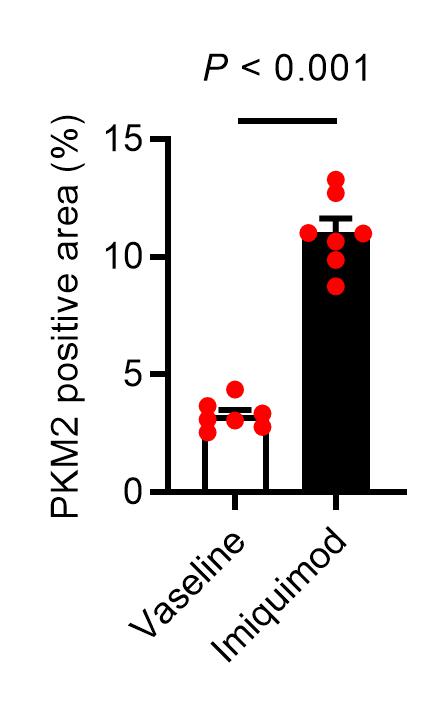


Figure S8 Quantitative results of PKM2 positive staining area percentage by immunohistochemistry in the skin of control mice or IMQ treatment mice. n=7.

Table S1. Baseline characteristics

| Baseline characteristic | Non psoriasis | Psoriasis |
| --- | --- | --- |
| N | 10 | 10 |
| Male | 5 | 5 |
| Age, y, mean (SD) | 47.7  (14.6) | 48 (14.9) |
| Psoriatic arthritis, n (%) | 0 | 3 (30%) |
| PASI, mean (SD) | 0 | 13.37 (0.92) |

PASI, Psoriasis Area and Severity Index.

Table S2 Statistical output

| Figure | Group | *t*-value | *P* value |
| --- | --- | --- | --- |
| 2B | Vaseline vs Imiquimod | 9.096 | <0.0001 |
| 2E | Vaseline vs Imiquimod | 5.577 | 0.0001 |
| 3B | GFP vs Flag-PKM2 | 5.245 | 0.0004 |
| 3C | GFP vs Flag-PKM2 | 3.610 | 0.0048 |
| 3E | Control siRNA vs PKM2 shRNA | 4.783 | 0.0007 |
| 3F | Control siRNA vs PKM2 shRNA | 2.713 | 0.0218 |
| 3G | Control siRNA vs PKM2 shRNA | 5.004 | 0.0005 |
| 3I | GFP vs Ad-PKM2 | 16.55 | <0.0001 |
| 3I | Control shRNA vs PKM2 shRNA | 78.13 | <0.0001 |
| 3J | GFP vs Ad-PKM2 | 11.12 | <0.0001 |
| 3J | Control shRNA vs PKM2 shRNA | 7.168 | <0.0001 |
| 4G | DMSO vs 2-DG | 6.649 | 0.0008 |
| 4G | DMSO vs Shikonin | 3.839 | 0.0400 |
| 4H | DMSO vs Shikonin | 6.139 | 0.0003 |
| 6B | Vaseline vs Imiquimod | 8.106 | <0.0001 |
| 6B | Imiquimod vs Imiquimod + 2-DG | 5.442 | 0.0004 |
| 6B | Imiquimod vs Imiquimod + Shikonin | 4.869 | 0.0005 |
| 6C | Vaseline vs Imiquimod | 5.577 | 0.0001 |
| 6C | Imiquimod vs Imiquimod + 2-DG | 4.892 | 0.0006 |
| 6C | Imiquimod vs Imiquimod + Shikonin | 2.541 | 0.0293 |
| 7C | Con vs EGF | 6.723 | 0.0007 |
| 7C | EGF vs EGF+AG-1478 | 11.00 | <0.0001 |
| 7D | Con vs EGF | 6.046 | 0.0018 |
| 7D | EGF vs EGF+AG-1478 | 9.661 | <0.0001 |
| 7F | Con vs EGF | 16.35 | <0.0001 |
| 7G | Con vs EGF | 3.747 | 0.0038 |
